# Supplementary material for: Genome of the world’s smallest flowering plant, Wolffia australiana, helps explain its specialized physiology and unique morphology
Source: Commun Biol. 2021 Jul 22;4:900. doi: 10.1038/s42003-021-02422-5 (PMC8298427; doi:10.1038/s42003-021-02422-5)
Supplement: Supplementary file 1 — Supplementary Figures [file 42003_2021_2422_MOESM1_ESM.pdf]

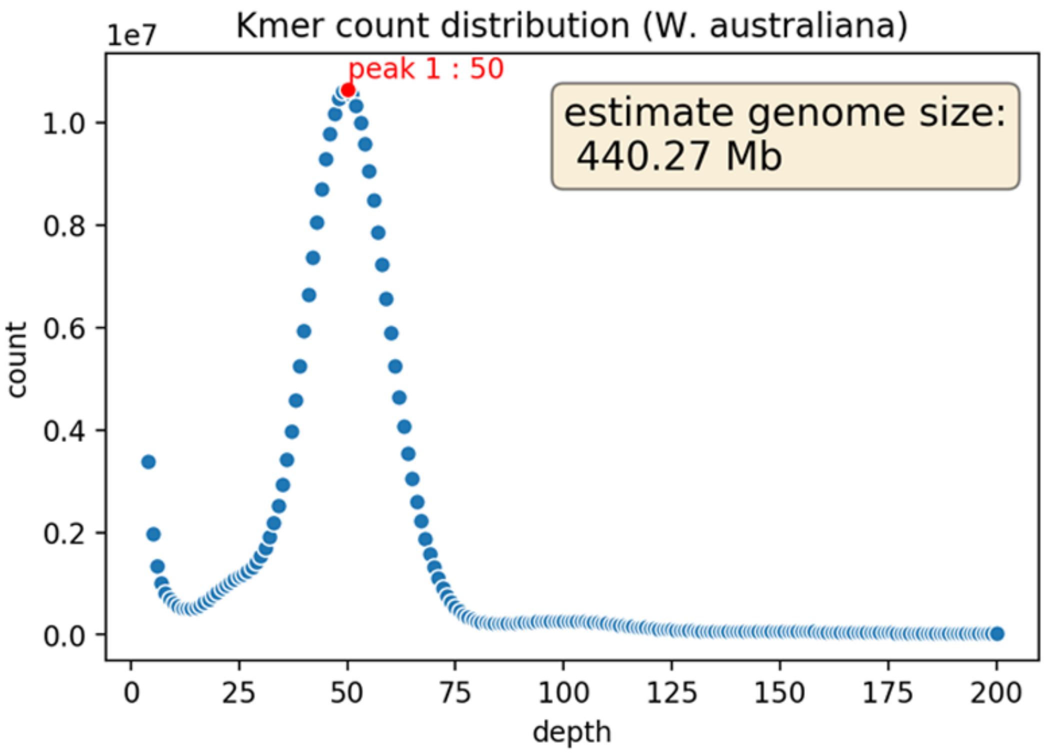

Supplementary Fig. 1. Kmer count distribution for estimating *W. australiana* genome size

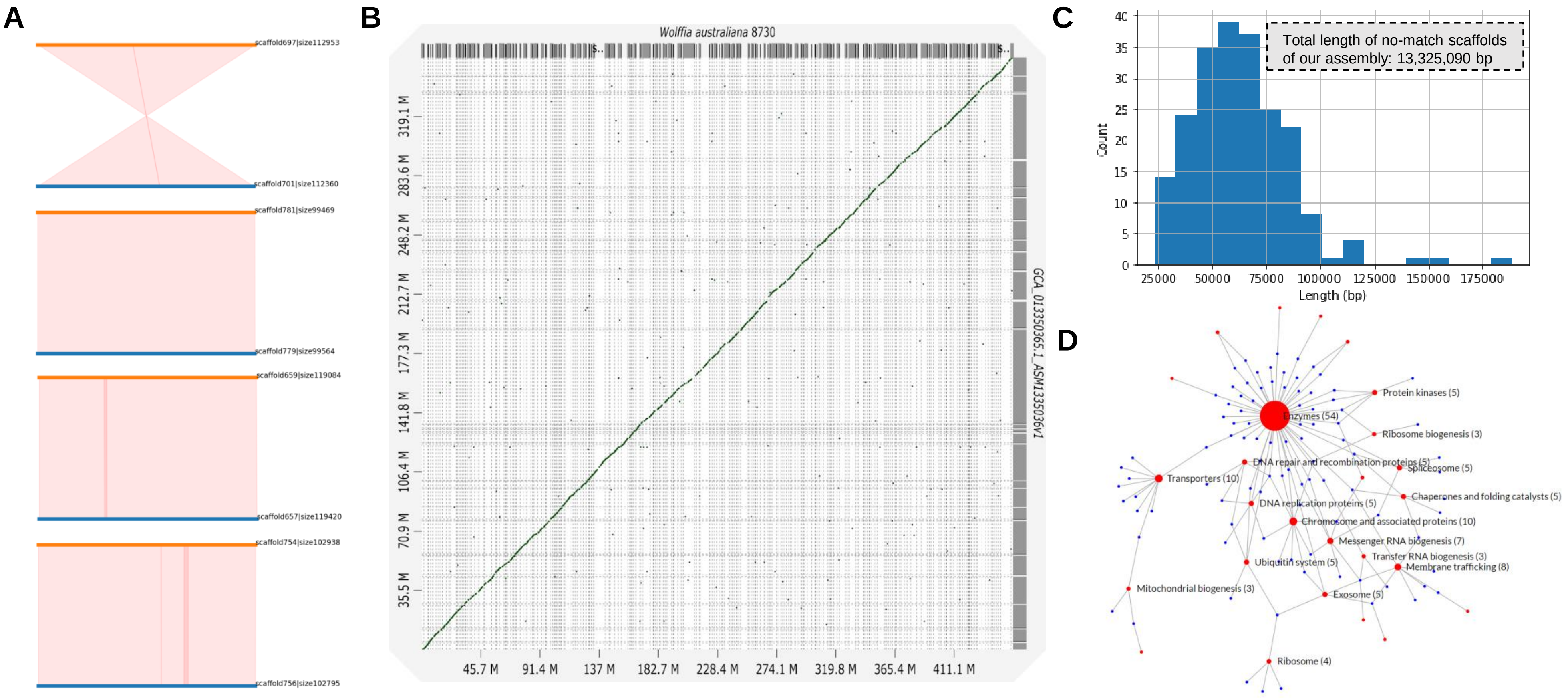

Supplementary Fig. 2. Quality assessment and curation of *W. australiana* genome assembly. **A**. The examples of the duplicated contigs in the *de novo* assembly. **B**. Comparison between our *W. australiana* 8730 genome assembly and Wa7733 assembly of Michael et. al. **C**. Scaffold length distribution of additional scaffolds of *W. australiana* 8730 compared to Wa7733. **D**. Kegg classification of the predicted genes that are present in additional scaffolds of our assembly.

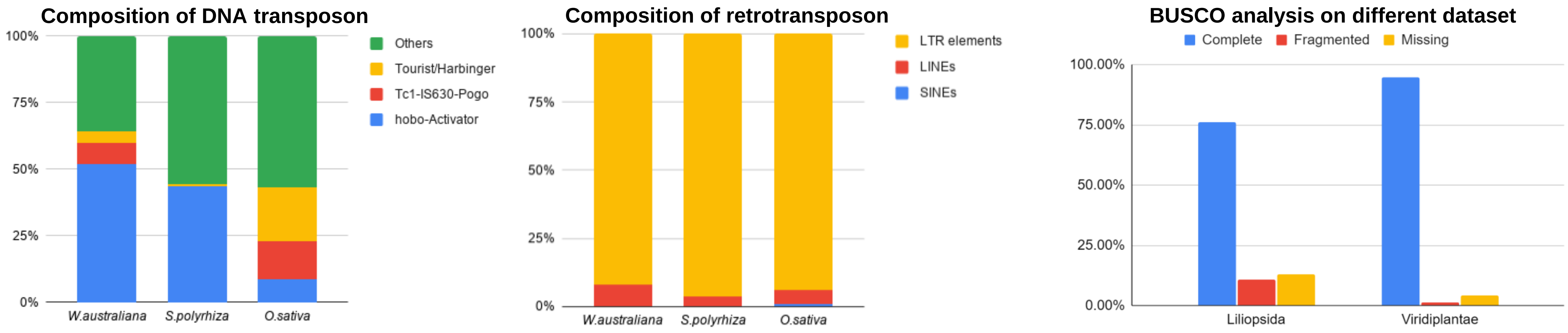

Supplementary Fig. 3. Repeat composition and predicted gene contents in *W. australiana* genome

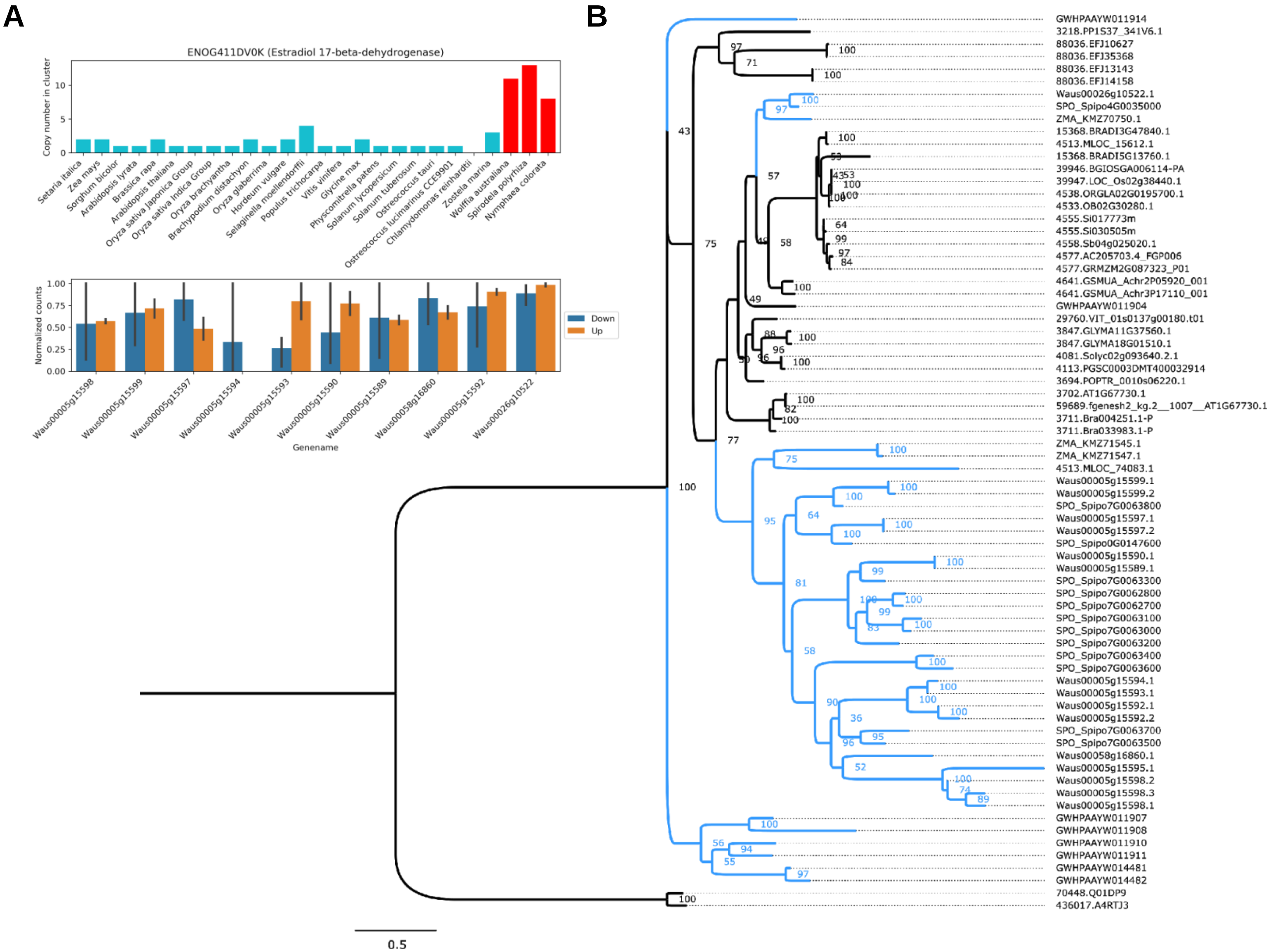

Supplementary Fig.4. Gene family evolution of estradiol 17-beta-dehydrogenase **A.** The copy number elevation of water floating plants and their gene expression at floating and submerged phase **B.** Gene tree of estradiol 17-beta-dehydrogenase; the blue lines indicate the water plants.

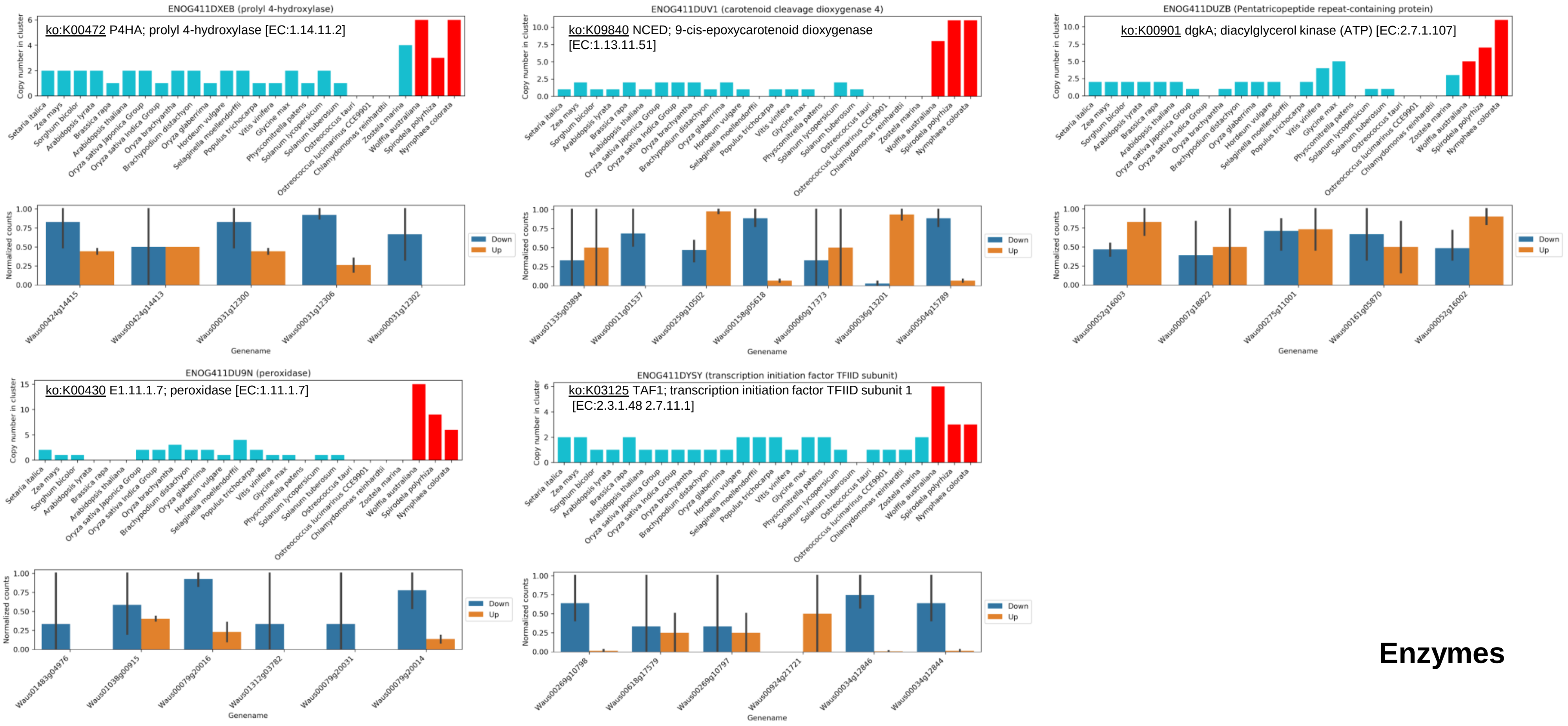

Supplementary Fig. 5. Expanded enzyme clusters of water floating plants with Kegg annotations. The upper panel shows the copy number in the cluster, and the bottom panel shows the gene expressions of the corresponding genes.

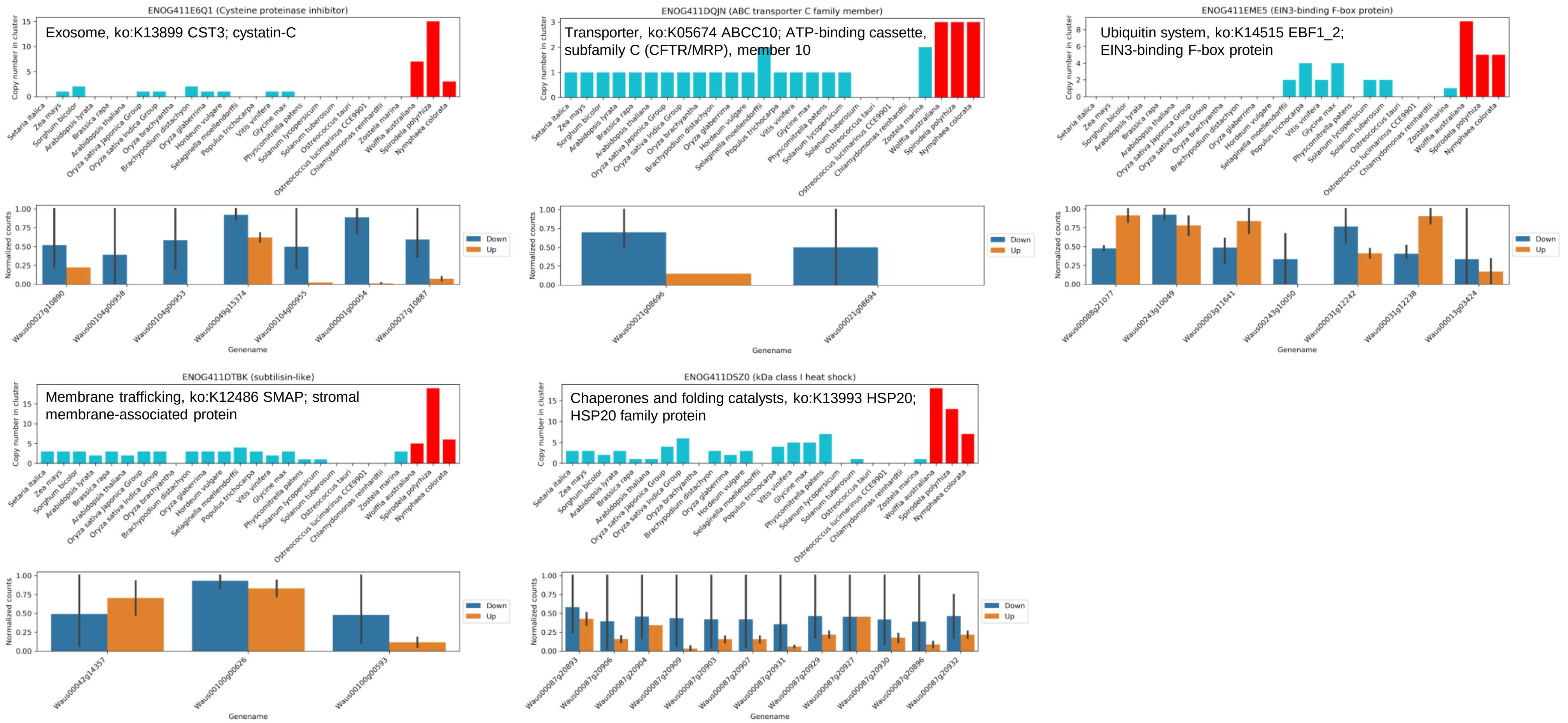

Supplementary Fig.6. Expanded non-enzyme clusters of water floating plants with Kegg annotations. The upper panel shows the copy number in the cluster, and the bottom panel shows the gene expressions of the corresponding genes.

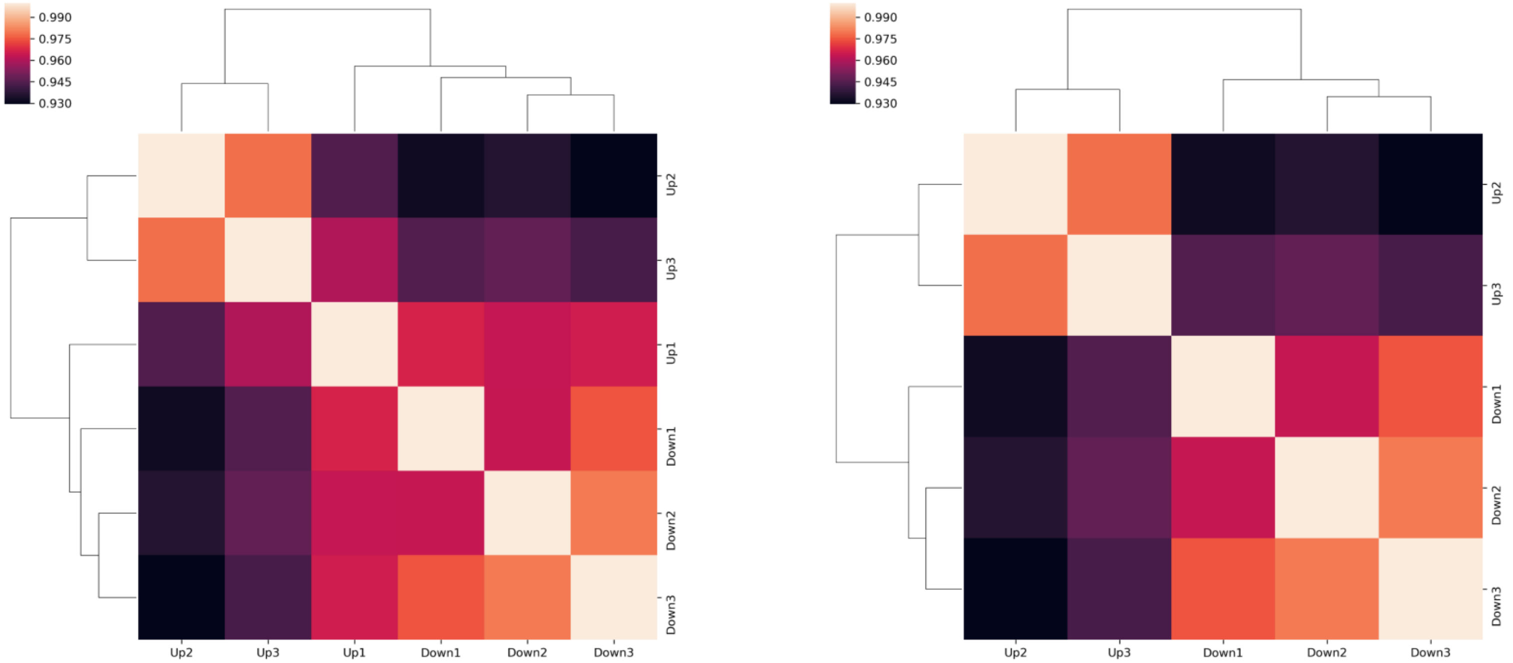

Supplementary Fig. 7. RNAseq sample correlation heatmap A. whole samples correlation plot shows incorrect clustering of "Up1" sample that possibly failed condition controls. B. After excluding "Up1" sample the heatmap shows clear clustering between the two phase, floating and submerged phase.

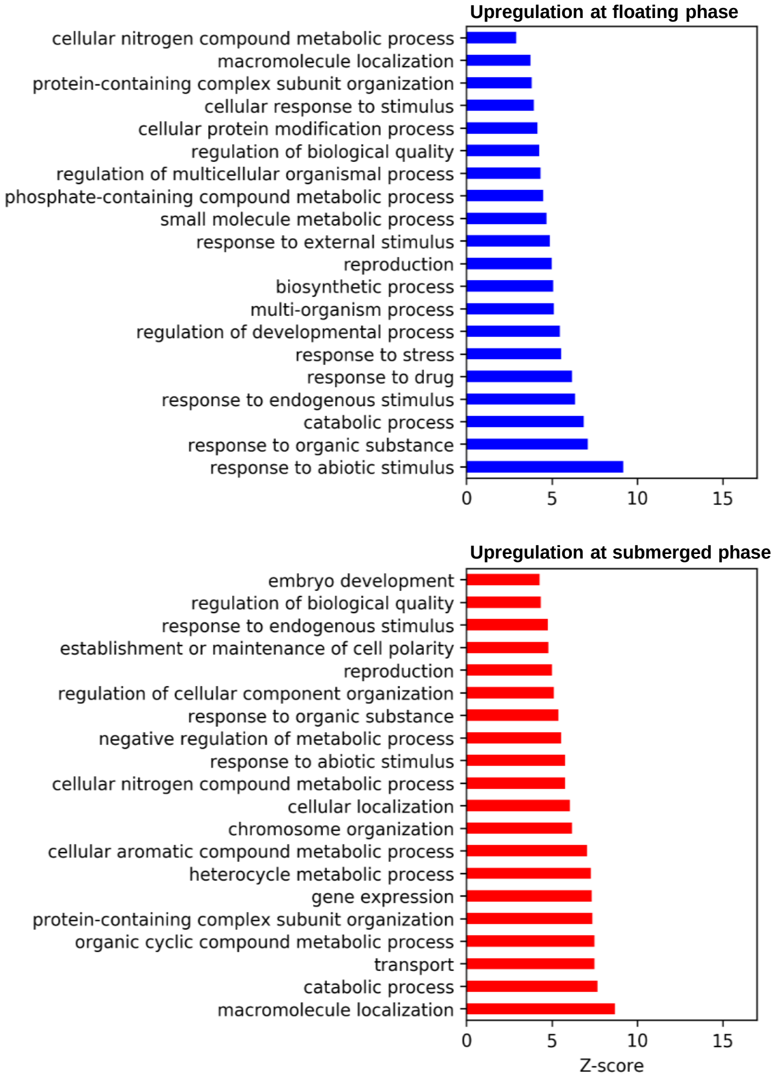

Supplementary Fig. 8. The GO enrichment analysis of the up-regulating gene set of floating phase (top) and up-regulating gene set of submerged phase (bottom)

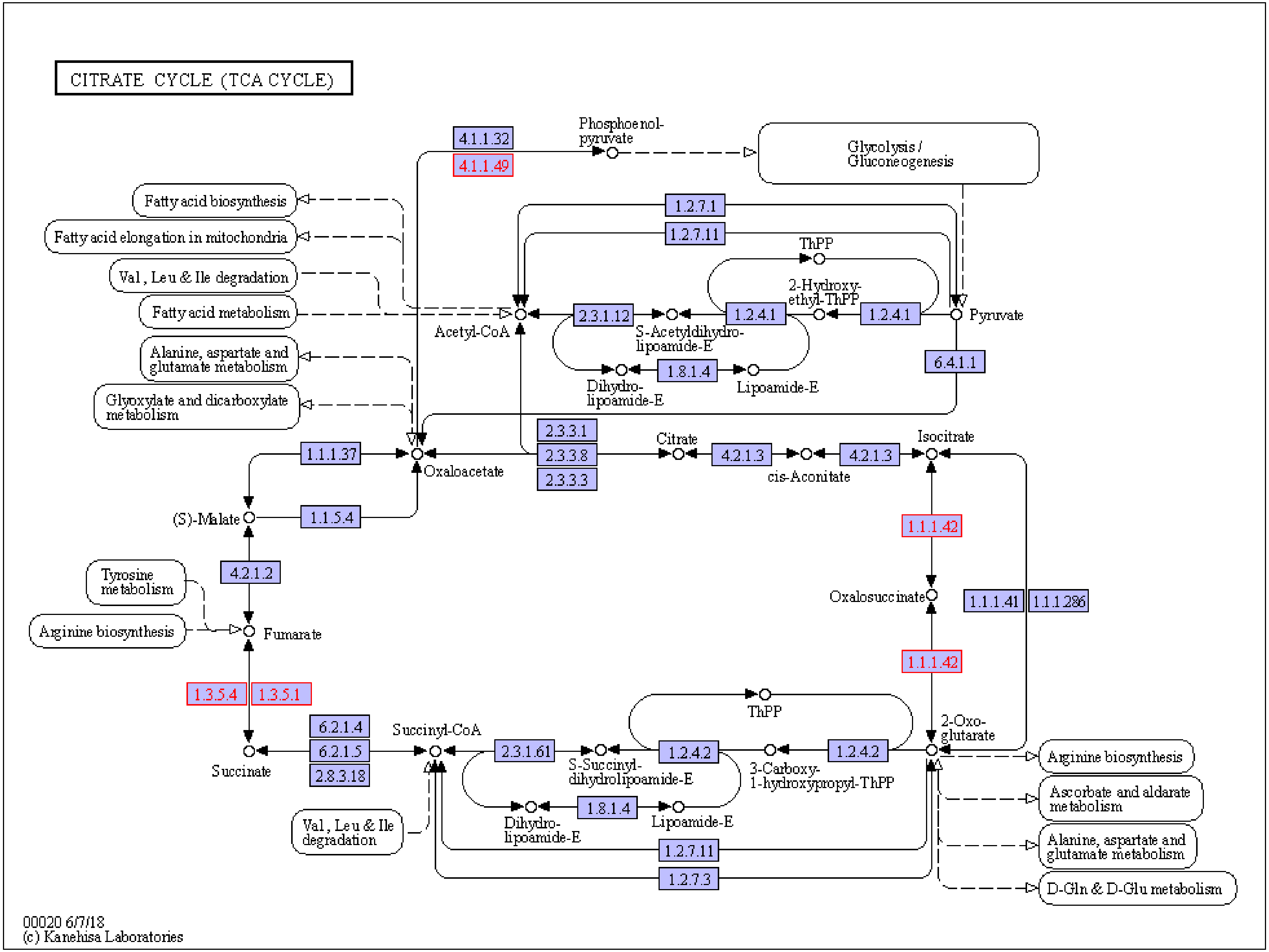

Supplementary Fig. 9. The context of upregulated genes in submerged phase in the pathway of TCA cycle
