# Supplementary material for: Genome of the world’s smallest flowering plant, Wolffia australiana, helps explain its specialized physiology and unique morphology
Source: Commun Biol. 2021 Jul 22;4:900. doi: 10.1038/s42003-021-02422-5 (PMC8298427; doi:10.1038/s42003-021-02422-5)
Supplement: Supplementary file 2 — Descriptions of Additional Supplementary Files [file 42003_2021_2422_MOESM2_ESM.pdf]

Descriptions of Additional Supplementary Files

Supplementary Data 1

Description: Sequencing amounts of various types of sequencing platform

Supplementary Data 2

Description: N50 statistics of assembly strategies

Supplementary Data 3

Description: Summary of detected repeat sequences

Supplementary Data 4. Gene family counts of *Viridiplantae*

Supplementary Data 5.

Description: *W. australiana* missing and over-representing Eggnog clusters

Supplementary Data 6.

Description: The presence and absence of stomata development related genes in the water plants

Supplementary Data 7.

Description: List of synteny blocks that are fractionated or missing in *W. australiana* genome

Supplementary Data 8.

Description: KaKs between *O. sativa* and *W. australiana*

Supplementary Data 9.

Description: KaKs between *O. sativa* and *S. polyrhiza*

Supplementary Data 10.

Description: Gene expression of *W. australiana* in floating and submerged phase

Supplementary Data 11.

Description: DEG statistics for the floating and submerged phases of *W. australiana*

(log2FoldChange > 0: Floating upregulation, log2FoldChange < 0: Submerged upregulation)

Supplementary Data 12.

Description: The significant DEG counts between the submerged and floating phases that are annotated by Kegg Orthology

Supplementary Data 13.

Description: VCF file resulted from remapping *W. australiana* reads to *W. australiana* genome
