# Supplementary material for: Genome of the world’s smallest flowering plant, Wolffia australiana, helps explain its specialized physiology and unique morphology
Source: Commun Biol. 2021 Jul 22;4:900. doi: 10.1038/s42003-021-02422-5 (PMC8298427; doi:10.1038/s42003-021-02422-5)
Supplement: Supplementary file 16 — Reporting Summary [file 42003_2021_2422_MOESM16_ESM.pdf]

Corresponding author(s): Hwan Su Yoon and Yang Jae Kang
Last updated by author(s): Jun 2, 2021

Reporting Summary

Nature Portfolio wishes to improve the reproducibility of the work that we publish. This form provides structure for consistency and transparency in reporting. For further information on Nature Portfolio policies, see our Editorial Policies and the Editorial Policy Checklist.

Statistics

For all statistical analyses, confirm that the following items are present in the figure legend, table legend, main text, or Methods section.

n/a	Confirmed	
☐	☒	The exact sample size (*n*) for each experimental group/condition, given as a discrete number and unit of measurement
☐	☒	A statement on whether measurements were taken from distinct samples or whether the same sample was measured repeatedly
☒	☐	The statistical test(s) used AND whether they are one- or two-sided<br>*Only common tests should be described solely by name; describe more complex techniques in the Methods section.*
☒	☐	A description of all covariates tested
☒	☐	A description of any assumptions or corrections, such as tests of normality and adjustment for multiple comparisons
☒	☐	A full description of the statistical parameters including central tendency (e.g. means) or other basic estimates (e.g. regression coefficient) AND variation (e.g. standard deviation) or associated estimates of uncertainty (e.g. confidence intervals)
☒	☐	For null hypothesis testing, the test statistic (e.g. *F*, *t*, *r*) with confidence intervals, effect sizes, degrees of freedom and *P* value noted<br>*Give P values as exact values whenever suitable.*
☒	☐	For Bayesian analysis, information on the choice of priors and Markov chain Monte Carlo settings
☒	☐	For hierarchical and complex designs, identification of the appropriate level for tests and full reporting of outcomes
☒	☐	Estimates of effect sizes (e.g. Cohen's *d*, Pearson's *r*), indicating how they were calculated

Our web collection on statistics for biologists contains articles on many of the points above.

Software and code

Policy information about availability of computer code

Data collection	1. Download RepeatModeler pipeline for repeat element analysis<br>http://www.repeatmasker.org/RepeatModeler<br>2. Download Repeat Library for repeat element analysis<br>http://www.girinst.org<br>3.Download cluster information for phylogenetic analysis<br>https://eggnog45.embl.de

Data analysis	1. Falcon assembler for assembly<br>https://github.com/PacificBiosciences/pb-assembly<br>2. BWA (Burrows-Wheeler Aligner) program for align DNA sequencing reads to genome assembly<br>http://bio-bwa.sourceforge.net/<br>3. Samtools for error correction of genoem assembly<br>https://github.com/samtools/samtools<br>4. Kallisto for align RNAseq reads to genome assembly<br>https://pachterlab.github.io/kallisto/<br>5. AUGUSTUS for gene-modeling<br>https://github.com/Gaius-Augustus/Augustus<br>6. BUSCO (Benchmarking Universal Single-Copy Orthologs) analysis for quality check of gene-models<br>https://busco.ezlab.org/v1/<br>7. KEGG annotations for gene analysis of metabolic pathways and functional annotations<br>https://www.kegg.jp/blastkoala/<br>8. Uniprot database information for search of protein functions<br>https://www.uniprot.org/

For manuscripts utilizing custom algorithms or software that are central to the research but not yet described in published literature, software must be made available to editors and reviewers. We strongly encourage code deposition in a community repository (e.g. GitHub). See the Nature Portfolio guidelines for submitting code & software for further information.

Data

Policy information about availability of data

All manuscripts must include a data availability statement. This statement should provide the following information, where applicable:

- Accession codes, unique identifiers, or web links for publicly available datasets
- A description of any restrictions on data availability
- For clinical datasets or third party data, please ensure that the statement adheres to our policy

NCBI BioProject accession number for genome assembly: PRJNA611905. The raw reads of W. australiana, including genome and transcriptome data, can be downloaded from NCBI SRA, BioProject accession number: PRJNA734041. The genome and gene information are freely shared at https://duckweeds.plantprofile.net/.

Field-specific reporting

Please select the one below that is the best fit for your research. If you are not sure, read the appropriate sections before making your selection.

☐ Life sciences ☐ Behavioural & social sciences ☒ Ecological, evolutionary & environmental sciences

For a reference copy of the document with all sections, see nature.com/documents/nr-reporting-summary-flat.pdf

Ecological, evolutionary & environmental sciences study design

All studies must disclose on these points even when the disclosure is negative.

Study description	We present the draft nuclear genome of Wolffia australiana generated using long-read PacBio sequence data and the 10x Genomics platform. Among Wolffia species, including W. brasiliensis (~800 Mb), W. globosa (~1.3 Gb), and W. arrhiza (~1.9 Gb),W. australiana has the smallest genome size (~400 Mb). Using transcriptome data to validate predicted gene models, we created a robust gene inventory for W. australiana. These new genome data were compared to published genomes from two duckweed sister species
Research sample	For plant tissue, we acquired Wolffia australiana 8730 from the Rutgers Duckweed Stock Cooperative (RDSC, http://www.ruduckweed.org/). The culture medium was composed of 0.5X SH salt, 1.5% sucrose and adjusted to pH 6. The condition of culture was in the temperature of 22oC with the light condition of 5000 lux.
Sampling strategy	[N/A] In this study, there was no experiment to determine sample-size.
Data collection	Using the Covaris G-tube, we generated 20 Kb fragments of genomic DNA according to the manufacturer's recommended protocol. We additionally used the AMpureXP bead purification system to eliminate small fragments. A total of 5 !g for each sample was used as input for the preparation of the sequencing library. The SMRTbell library was built using the SMRTbell ® Express Template Preparation Kit (101-357-000). The SMRTbell library was sequenced using SMRT cells (Pacific Biosciences) and Sequel Sequencing Kit v3.0. Total 1×10-hour real-time sequencing were recorded for each SMRT Cell 1M v3 using the sequel (Pacific Biosciences, PacBio) sequencing platform.
Timing and spatial scale	[N/A] In this study, there was no experiment to require timing and spatial scale.
Data exclusions	[N/A]
Reproducibility	[N/A] In this study, there was no experiment related to reproducibility.
Randomization	[N/A] In this study, there was no experiment consider to randomization.

Blinding	[N/A] There was no blinding information in this study.

Did the study involve field work? ☐ Yes ☒ No

Reporting for specific materials, systems and methods

We require information from authors about some types of materials, experimental systems and methods used in many studies. Here, indicate whether each material, system or method listed is relevant to your study. If you are not sure if a list item applies to your research, read the appropriate section before selecting a response.

Materials & experimental systems

n/a	Involved in the study
☒	☐ Antibodies
☒	☐ Eukaryotic cell lines
☒	☐ Palaeontology and archaeology
☒	☐ Animals and other organisms
☒	☐ Human research participants
☒	☐ Clinical data
☒	☐ Dual use research of concern

Methods

n/a	Involved in the study
☒	☐ ChIP-seq
☒	☐ Flow cytometry
☒	☐ MRI-based neuroimaging
